# Strategy Comparison of Endoscopic Ultrasound-Guided Gallbladder Drainage to Percutaneous Transhepatic Gallbladder Drainage, Following Failed Emergent Endoscopic Transpapillary Gallbladder Drainage

**DOI:** 10.3390/jcm12227034

**Published:** 2023-11-10

**Authors:** Ryota Sagami, Kazuhiro Mizukami, Takao Sato, Hidefumi Nishikiori, Kazunari Murakami

**Affiliations:** 1 Department of Gastroenterology, Oita San-ai Medical Center, 1213 Oaza Ichi, Oita 870-1151, Japan; sagami1985@yahoo.co.jp (R.S.); iwataiwata18@yahoo.co.jp (T.S.); nikki@san-ai-group.org (H.N.); 2 Department of Gastroenterology, Faculty of Medicine, Oita University, 1-1 Idaigaoka, Hasamacho, Yufu 879-5593, Japan; murakam@oita-u.ac.jp

**Keywords:** acute cholecystitis, endoscopic gallbladder drainage, endoscopic transpapillary gallbladder drainage, endoscopic ultrasound-guided gallbladder drainage, percutaneous transhepatic gallbladder drainage

## Abstract

Endoscopic transpapillary gallbladder drainage (ETGBD) is recommended for patients with acute cholecystitis at high risk for surgery/percutaneous transhepatic gallbladder drainage (PTGBD). Endoscopic ultrasound-guided gallbladder drainage (EUS-GBD) has higher success and mortality rates than ETGBD. Optimal endoscopic drainage remains controversial. Patients with moderate/severe acute cholecystitis and high risk for surgery/PTGBD who underwent ETGBD were enrolled. In the new-ETGBD (N-ETGBD)/traditional-ETGBD (T-ETGBD) strategy, patients in whom the initial ETGBD failed underwent rescue-EUS-GBD in the same endoscopic session/rescue-PTGBD, respectively. Therapeutic outcomes were compared. Patients who could not undergo rescue-EUS-GBD/PTGBD owing to poor general conditions received conservative treatment. Technical success was defined as successful ETGBD or successful rescue-EUS-GBD/PTGBD. Forty-one/forty patients were enrolled in the N-ETGBD/T-ETGBD groups, respectively. The N-ETGBD group had a higher, though non-significant, technical success rate compared to the T-ETGBD group (97.6 vs. 90.0%, *p* = 0.157). The endoscopic technical success rate was significantly higher in the N-ETGBD than in the T-ETGBD group (97.6 vs. 82.5%, *p* = 0.023). The clinical success/adverse event rates were similar between both groups. The hospitalization duration was significantly shorter in the N-ETGBD than in the T-ETGBD group (6.6 ± 3.9 vs. 10.1 ± 6.4 days, *p* < 0.001). ETGBD with EUS-GBD as a rescue backup may be an ideal hybrid drainage for emergency endoscopic gallbladder drainage in high-risk surgical patients.

## 1. Introduction

Emergency cholecystectomy for patients with multiple severe comorbidities may increase mortality and the risk for further surgery [1]. Traditionally, percutaneous transhepatic gallbladder drainage (PTGBD) is the drainage method of choice for high-risk surgical patients with acute cholecystitis [2,3]. However, PTGBD is not recommended for patients undergoing antithrombotic therapies, those with an anatomically inaccessible gallbladder, or those at risk for tube self-removal owing to advanced age [4,5,6]. In such cases, endoscopic transpapillary gallbladder drainage (ETGBD), which has a relatively high success rate, is usually considered when skilled endoscopists are available [2,7]. A recent randomized controlled trial showed that endoscopic ultrasound-guided gallbladder drainage (EUS-GBD) had better outcomes than PTGBD in high-risk surgical patients [8]. In addition, the adverse event rate was higher and the length of hospitalization was longer for PTGBD than for EUS-GBD [8,9,10,11].

In recent years, the reported technical and clinical success rates of EUS-GBD using lumen-apposing/self-expandable metal stents have been higher than those of ETGBD [12,13,14]. However, EUS-GBD is sometimes associated with severe or fatal adverse events [14,15,16]. Moreover, metal stents remain unavailable in several countries [17]. Conversely, ETGBD has a similar incidence rate of adverse events but a lower mortality rate than EUS-GBD [14,18] and can be performed using normal plastic stents. Thus, establishing the optimal procedure for endoscopic gallbladder drainage (EGBD) remains controversial.

Patients with moderate/severe acute cholecystitis who undergo ETGBD as the first-line treatment and experience technical failure require high-risk PTGBD or high-risk conservative treatment without aggressive drainage. We hypothesized that the new ETGBD strategy (N-ETGBD; patients undergo ETGBD as the first-line drainage, and if ETGBD fails, they undergo rescue-EUS-GBD in the same session) may be clinically more successful than the traditional ETGBD strategy (T-ETGBD; patients undergo ETGBD as the first-line drainage, and if ETGBD fails, they undergo high-risk rescue-PTGBD). Conversely, whether undergoing EUS-GBD after failed ETGBD in the same session increases the adverse event/mortality rate is uncertain.

Therefore, this study aimed to evaluate the clinical course and safety of N-ETGBD compared with those of T-ETGBD as an emergent drainage method for high-risk surgical patients with acute cholecystitis.

## 2. Materials and Methods

### 2.1. Eligibility Criteria

This retrospective study was designed and conducted in the two participating facilities in accordance with the Declaration of Helsinki, and was registered in the University Hospital Medical Network Clinical Trials Registry (UMIN000047977) after receiving approval from our Institutional Review Board (IRB Protocol Number: 021001K). The requirement of obtaining informed consent was waived because of the retrospective nature of the study.

Between January 2018 and October 2022, patients who initially underwent ETGBD because they had moderate/severe acute cholecystitis and were at high-risk for surgery/PTGBD were included. The reasons for being at high-risk for surgery/PTGBD were as follows: advanced age (>85 years), continuous single antithrombotic therapy without aspirin/multiple antithrombotic therapies, severe comorbidities, and compromised physical conditions. The severity of acute cholecystitis was determined according to the Tokyo Guidelines 2018 [19]. The severity of the patients’ physical condition, considering comorbidities and indications for surgery, was determined according to the American Society of Anesthesiologists physical status classification system [20].

Patients who underwent initial gallbladder drainage other than ETGBD and those who had undergone prior gallbladder drainage were excluded. Patients with cholecystitis accompanying choledocholithiasis were also excluded, as the condition of most patients with cholecystitis caused by choledocholithiasis can be improved after common bile duct treatment [5].

### 2.2. Drainage Strategy for N-ETGBD and T-ETGBD

All participating endoscopists had previously performed >500 endoscopic retrograde cholangiopancreatography and >50 ETGBD procedures.

The drainage strategy of choice for emergent initial ETGBD was changed from T-ETGBD to N-ETGBD after the performance of 10 elective EUS-GBD procedures at the facility by the operators. The T-ETGBD group included all patients who underwent ETGBD from January 2018 to March 2019 (when PTGBD was used as a rescue drainage). The N-ETGBD group included all patients who underwent ETGBD from April 2019 to October 2022 (when EUS-GBD was used as a rescue drainage).

**Figure 1:**
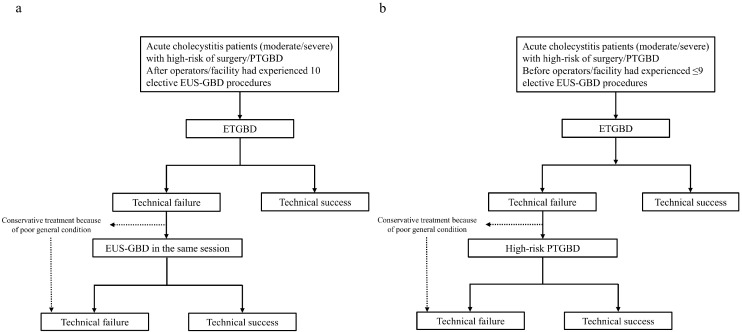
Treatment strategies of new endoscopic transpapillary gallbladder drainage (N-ETGBD) and traditional endoscopic transpapillary gallbladder drainage (T-ETGBD). (**a**) In the N-ETGBD strategy, EUS-GBD was performed following failed ETGBD in the same session. (**b**) In the T-ETGBD strategy, PTGBD was attempted following failed ETGBD.

The N-ETGBD and T-ETGBD strategies are presented in Figure 1. In the N-ETGBD strategy, patients in whom the initial ETGBD failed underwent rescue-EUS-GBD in the same endoscopic session (Figure 1a). Patients who underwent rescue-EUS-GBD failure received conservative treatment without additional PTGBD. In the T-ETGBD strategy, patients in whom the initial ETGBD failed underwent high-risk PTGBD (Figure 1b). Patients for whom EUS-GBD/PTGBD could not be attempted because of their poor general conditions, including multiple antithrombotic therapies, underwent conservative treatment.

### 2.3. ETGBD Technique

**Figure 2:**
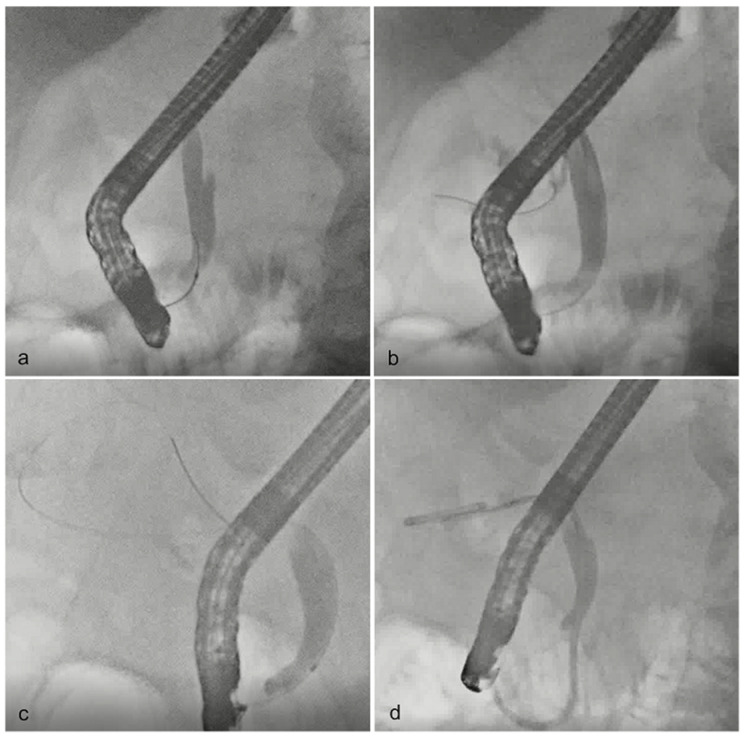
Techniques of endoscopic transpapillary gallbladder drainage. (**a**) After successful cannulation into the common bile duct, the cystic duct orifice was clarified using a contrast agent. (**b**) When cystic duct enhancement was not achieved, a guidewire was used to identify the orifice and was manipulated into the spiral cystic duct. (**c**) A guidewire was deeply inserted into the gallbladder. (**d**) A double pigtail-type plastic stent was placed from the gallbladder to the duodenum.

A duodenoscope (JF 260V; Olympus Medical Systems, Tokyo, Japan) was inserted into the duodenum and cannulated across the duodenal papilla into the common bile duct using an endoscopic retrograde cholangiopancreatography catheter (Swish; Boston Scientific, Marlborough, MA, USA) and a 0.025-inch guidewire (M-through; Asahi Intecc, Aichi, Japan) (Figure 2a). After common bile duct cannulation and contrast agent injection, the cystic duct was enhanced, and cannulation to the gallbladder was performed, aiming for an enhanced branch point of the cystic duct from the common bile duct. When the cystic duct was not enhanced, the guidewire was caught and used as a marker for identifying the orifice (Figure 2b). After protective manipulation and deep insertion of the guidewire through the intricate spiral cystic duct (Figure 2c), the catheter was deeply inserted, and infected bile was aspirated as much as possible. A contrast agent was injected to confirm the gallbladder lumen. Subsequently, a plastic stent (double pigtail-type stent, 6-Fr, 12 or 10 cm; Hanaco Medical, Saitama, Japan) was placed from the gallbladder to the duodenum (Figure 2d). Completion of this series of procedures was considered a technical success.

### 2.4. EUS-GBD Technique

**Figure 3:**
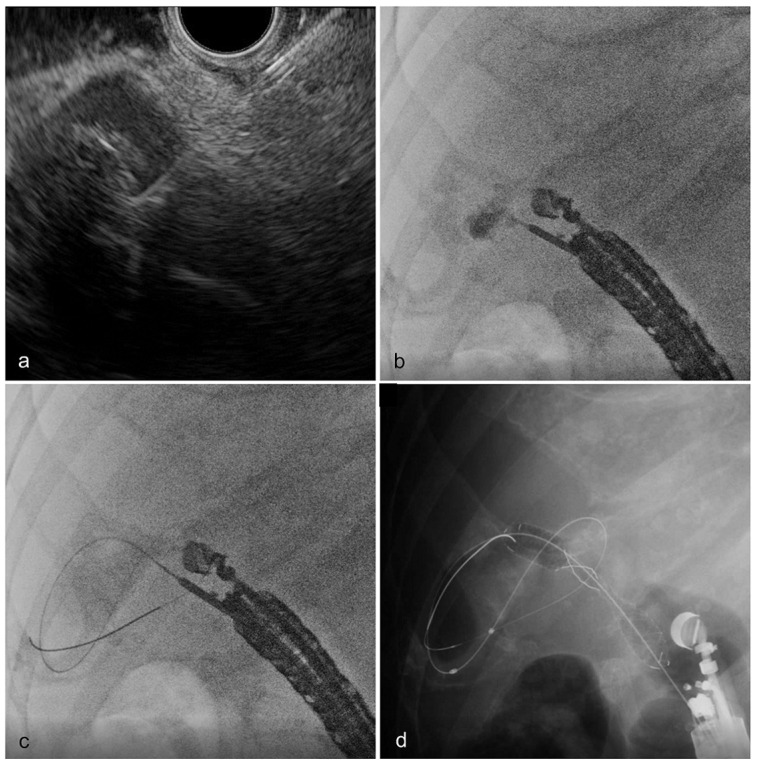
Techniques of endoscopic ultrasound-guided gallbladder drainage. (**a**) The gallbladder was detected using endoscopic ultrasound and punctured using a 19 G fine aspiration needle, while avoiding conspicuous blood vessels. (**b**) After adequate aspiration of infected bile, the contrast agent was administered through the needle. (**c**) After deep insertion of a guidewire into the gallbladder, the puncture tract was dilated using a dilator along the inserted guidewire. (**d**) A fully covered dumbbell-type metal stent was placed from the gallbladder to the gastrointestinal tract.

When ETGBD was technically unsuccessful, the duodenoscope was removed, and an echoendoscope (GF-UCT260; Olympus Medical Systems) paired with ultrasound processors (ProSound SSD F75/ARIETTA; Aloka, Tokyo, Japan) was inserted into the stomach antrum or duodenal bulb. The puncture lumen (stomach or duodenum) was determined by operators after considering patient-specific anatomy and the proximity of the gallbladder to the lumen. The gallbladder was detected with EUS in the color Doppler mode and punctured using a 19 G fine aspiration needle (EZ-shot 3 Plus; Olympus) while avoiding conspicuous blood vessels (Figure 3a). After adequate aspiration of infected bile, a contrast agent was injected through the needle (Figure 3b). The puncture tract was then dilated using a dilator (Ren 4 mm; Kaneka, Yokohama, Japan or Fine-025; Medicos Hirata, Osaka, Japan) along the inserted guidewire (Figure 3c). Subsequently, a fully covered dumbbell-type self-expandable metal stent (M-Intraductal 10 mm, 7 cm; Medicos Hirata) was placed from the gallbladder to the gastrointestinal tract (Figure 3d). Completion of this series of procedures was considered a technical success.

### 2.5. Definition and Study Outcome

To evaluate the overall strategy of N-ETGBD/T-ETGBD, technical success in the N-ETGBD/T-ETGBD groups was defined as successful performance of ETGBD or successful rescue-EUS-GBD/PTGBD following a failed ETGBD procedure. Patients from both groups who could not undergo any rescue procedure owing to their poor general conditions underwent conservative treatment and were considered technical failure cases.

The clinical success rate was calculated from the following cases: cases with technical success and cases receiving conservative treatment after technical failure of N-ETGBD/T-ETGBD. Clinical success was defined as an improvement in cholecystitis without an emergent lifesaving surgery/drainage other than with N-ETGBD or T-ETGBD strategies based on improvements in objective clinical symptoms and the levels of inflammatory parameters on blood examination. The severity of adverse events was determined according to the American Society of Gastrointestinal Endoscopy classification [21].

We also evaluated whether technical success was achieved with endoscopic procedures alone. Endoscopic technical success was defined as the technical success of ETGBD or rescue-EUS-GBD after ETGBD failure, and this was evaluated in both strategies.

The success rates (technical, clinical, and endoscopic technical) of N-ETGBD and T-ETGBD as emergent gallbladder drainage strategies for high-risk surgical patients were compared. Furthermore, post-procedural details, procedure-related adverse event/mortality rates, time until normalization of the white blood cell (WBC) count, and length of hospital stay (days until possible discharge) were compared between the groups.

### 2.6. Statistical Analyses

We compared patient characteristics and therapeutic outcomes between the T-ETGBD and N-ETGBD groups. Continuous variables are presented as means ± standard deviations, depending on the normality of the distribution. Categorical parameters were compared using the chi-square or Fisher’s exact test. These statistical analyses were conducted using Statistical Package for Social Sciences version 28.0 (IBM Corp., Armonk, NY, USA). Statistical significance was set at *p* < 0.05.

## 3. Results

We enrolled 41 and 40 patients in the N-ETGBD and T-ETGBD groups, respectively. Table 1 summarizes the baseline characteristics of the patients. No significant differences were noted between the groups. Table 2 summarizes the therapeutic outcomes of both groups. The N-ETGBD group had a higher, though non-significant, technical success rate compared with the T-ETGBD group (97.6% vs. 90.0%, *p* = 0.157). The endoscopic technical success rate was significantly higher with N-ETGBD than with T-ETGBD (97.6% vs. 82.5%, *p* = 0.023). The clinical success rates of both groups were almost the same.

In the N-ETGBD group, eight patients underwent rescue-EUS-GBD following unsuccessful ETGBD in the same session, and only one patient had gallbladder puncture failure owing to multiple stones in the gallbladder body and neck. The remaining patients underwent rescue-EUS-GBD with technical success.

No significant difference was noted in the incidence rate of adverse events between the two groups. Adverse events included mild pancreatitis and cholangitis, with no procedure-related fatal events occurring in either group. All adverse events of pancreatitis in the T-ETGBD group were associated with ETGBD. No significant difference was noted in the time to normalization of the WBC count. Finally, the length of hospitalization was significantly shorter in the N-ETGBD group than in the T-ETGBD group (6.6 ± 3.9 vs. 10.1 ± 6.4 days, *p* < 0.001). Finally, 12 patients who achieved technical success with ETGBD underwent elective cholecystectomy without any specific adverse events.

## 4. Discussion

This study examined the efficacy and safety of N-ETGBD compared with those of T-ETGBD. N-ETGBD had a higher, though non-significant, technical success rate of >97%. The endoscopic technical success rate was significantly higher in the N-ETGBD group than in the T-ETGBD group. No significant difference was noted in the incidence rate of adverse events between the groups, and no procedure-related fatal events occurred in either group.

In addition, N-ETGBD was associated with a shorter duration of hospitalization. One reason for this might have been that the rescue treatment after failed ETGBD could be performed in the same session. Additionally, cases with technical failure in the T-ETGBD group might have required a longer treatment period with conservative treatment or PTGBD. Therefore, the comprehensive follow-up period after N-ETGBD might be shorter than that after T-ETGBD. Comprehensively, the N-ETGBD strategy could provide effective and safe treatment in a single endoscopic session.

Cholecystectomy is a first-line treatment for acute cholecystitis. However, emergent surgery, including laparoscopic cholecystectomy, for patients with multiple severe comorbidities may increase mortality and the risk for further surgery [1]. PTGBD is traditionally recommended for such high-risk surgical patients as a less invasive treatment [2,3]. However, PTGBD tubes are at risk of being removed by older patients, especially those with dementia, and PTGBD is also not recommended for those with an anatomically inaccessible gallbladder or ascites [4,5]. PTGBD has a high risk of bleeding-related adverse events owing to needle puncture through the liver. Therefore, PTGBD is also not recommended for patients receiving antithrombotic therapy other than single aspirin therapy [6]. In addition, the fact that PTGBD tube removal is impossible until fistula formation (at least 2 weeks) increases the duration of hospitalization [22].

ETGBD is useful for high-risk patients requiring surgery/PTGBD [4,5], and it has relatively high technical and clinical success rates of 83.0% and 88.1%, respectively, as reported previously [16]. In addition, ETGBD has a lower adverse event rate and shorter length of hospitalization than PTGBD [9,10]. However, ETGBD presents with some technical difficulties, including difficulty in locating the cystic duct orifice when the cystic duct is not well contrasted or handling selective cystic duct cannulation through the guidewire into the gallbladder owing to calculus, malignant obstruction, or tortuosity [23,24]. Intraductal ultrasonography or cholangioscopy may help seek and cannulate the cystic duct orifice [25,26]; however, standardization of ETGBD has not yet been established.

EUS-GBD also has high clinical efficacy for patients with acute cholecystitis, with technical and clinical success rates of 95.3% and 96.7%, respectively [16]. Recent studies have shown that EUS-GBD had technical and clinical success rates similar or superior to those of PTGBD [8,18] and that EUS-GBD was superior to ETGBD [14,18]. Another study revealed that EUS-GBD had a significantly higher clinical success rate than PTGBD/ETGBD and that the incidence rates of adverse events of these three procedures were similar [16]. In addition, EUS-GBD requires a shorter duration of hospitalization than PTGBD [11]. Thus, EUS-GBD has been recommended as first-line EGBD for high-risk surgical patients with acute cholecystitis [3].

Typical adverse events, such as bleeding and perforation with incidence rates of 4.3% and 3.7%, respectively, have been reported with EUS-GBD [16]. ETGBD has a 5.1% risk of pancreatitis as the main adverse event, and the overall adverse event rates of EUS-GBD and ETGBD are reportedly similar (12.2% vs. 12.6%, *p* = 0.32) [16]. However, the overall bleeding adverse event rate of ETGBD is reported to be 0.65%, which is significantly lower than that of EUS-GBD [15]. Therefore, ETGBD may be the best option for patients on continuous antithrombotic therapy or with bleeding tendency [4,5,15].

In addition, bleeding/perforation adverse events associated with EUS-GBD sometimes become fatal; an all-cause mortality rate of 26% has been reported [16,27]. Meanwhile, ETGBD has the lowest mortality rate when compared with EUS-GBD and PTGBD [18]. Thus, ETGBD may be safer than EUS-GBD. In addition, metal stents used for EUS-GBD remain unavailable in several countries [17]. Depending on the facility, ETGBD with rescue-EUS-GBD as a backup treatment could be the optimal first-line EGBD strategy, although it is necessary to prepare for the potential adverse events of acute cholecystitis treatment including pseudoaneurysm bleeding and its subsequent symptoms, stent migration, and late perforation [15,28,29,30].

In this study, N-ETGBD achieved high technical and clinical success rates and reduced the length of hospitalization, with the active introduction of EUS-GBD in difficult cases of ETGBD. N-ETGBD may be a hybrid procedure with a low fatality rate, similar to that of ETGBD, and a high technical/clinical success rate, similar to that associated with EUS-GBD. The clinical implications of this combination strategy suggest that it may be an ideal drainage procedure for high-risk surgical patients with acute cholecystitis.

This study had some limitations. First, this study revealed better clinical efficacy of N-ETGBD compared to that of T-ETGBD. However, this study had a relatively small sample size and there was no statistical significance in the technical and clinical outcomes. Patients underwent N-ETGBD relatively safely with endoscopic simplicity; however, the procedure might be more complicated when compared to PTGBD. In addition, we focused on the efficacy of the whole strategy of N-ETGBD/T-ETGBD. However, we should essentially focus on only patients undergoing rescue-EUS-GBD or rescue-PTGBD after failed ETGBD. Evaluation of a larger number of patients is warranted.

Second, we only discussed the efficacy of N-ETGBD as an emergency gallbladder drainage method, while the management of deployed stents and long-term outcomes were not analyzed. The long-term recurrence rate of cholecystitis or cholangitis is reportedly higher with ETGBD than with EUS-GBD (12.4% vs. 3.2%) [31], and a recent meta-analysis reported a lower recurrence rate following EUS-GBD than following ETGBD or PTGBD [18]. Therefore, the long-term outcomes of N-ETGBD should be evaluated in the future.

In addition, emergent cholecystectomy/PTGBD for high-risk patients is sometimes performed in actual clinical practice. We should consider comparing our ETGBD strategies and emergent cholecystectomy/PTGBD for high-risk patients to establish further evidence. Further, large-scale, long-term, observational studies are required.

## 5. Conclusions

In conclusion, N-ETGBD strategy with a high technical success rate of >97%, significantly shortened duration of hospitalization than the T-ETGBD strategy, with similar results in technical success and incidence rates of adverse events. Thus, the rescue strategy of ETGBD with EUS-GBD as a backup may be an ideal hybrid drainage method for emergent EGBD in high-risk surgical patients.

## Figures and Tables

**Table 1:** Patients’ baseline characteristics.

	N-ETGBD (n = 41)	T-ETGBD (n = 40)	*p*-Value
Age, mean ± SD (years)	82.0 ± 8.9	74.9 ± 10.7	0.863
Male sex, n (%)	24 (58.5)	26 (65.0)	0.550
Severity of cholecystitis, n (%)			0.591
Moderate	33 (80.5)	34 (85.0)	
Severe	8 (19.5)	6 (15.0)	
WBC count, mean ± SD (×10^3^/μL)	15.0 ± 7.0	12.5 ± 5.8	0.125
CRP, mean ± SD (mg/dL)	15.8 ± 10.6	11.7 ± 9.8	0.557
Gallbladder stone	37 (90.2)	35 (87.5)	0.694
Main comorbidities, n (%)	38 (92.7)	35 (87.5)	0.434
Heart disease	23 (56.1)	19 (47.5)	0.439
Non-cardiac vascular disease	13 (31.7)	15 (37.5)	0.584
Chronic kidney disease/liver cirrhosis	3 (7.3)	2 (5.0)	0.665
Diabetes	8 (20.0)	8 (20.0)	0.956
Hypertension	13 (31.7)	20 (50.0)	0.094
ASA classification, n (%)			0.708
ASA I/II	25 (61.0)	26 (65.0)	
ASA III	16 (39.0)	14 (35.0)	
Reasons for high surgical/PTGBD risk, n (%)			0.613
ASA III	9 (22.0)	6 (15.0)	
Continuous antithrombotic therapy	12 (29.3)	11 (27.5)	
Advanced age (≥85 years)	15 (36.6)	14 (35.0)	

ASA, American Society of Anesthesiologists; CRP, C-reactive protein; N-ETGBD, rescue-endoscopic ultrasound-guided gallbladder drainage; PTGBD, percutaneous transhepatic gallbladder drainage; SD, standard deviation; T-ETGBD, rescue-percutaneous transhepatic gallbladder drainage; WBC, white blood cell.

**Table 2:** Therapeutic outcomes.

	N-ETGBD (n = 41)	T-ETGBD (n = 40)	*p*-Value
Technical success rate, n (%)	40 (97.6)	36 (90.0)	0.157
Clinical success rate, n (%)	39 (95.1)	38 (95.0)	0.980
Endoscopic technical success rate, n (%)	40 (97.6)	33 (82.5)	0.023
Adverse event rate, n (%)	3 (7.3)	2 (5.0)	0.665
Mild pancreatitis	2 (4.9)	2 (5.0)	
Cholangitis	1 (2.4)	0 (0)	
Additional treatment following unsuccessful ETGBD	8 (19.5)	7 (17.5)	0.390
EUS-GBD	8 (19.5)	0 (0)	
High-risk PTGBD	0 (0)	3 (7.5)	
High-risk conservative treatment (poor general condition including multiple antithrombotic therapies)	0 (0)	4 (10.0)	
Time until normalization of WBC count, mean ± SD (days)	3.7 ± 3.1	3.5 ± 3.3	0.267
Length of hospitalization, mean ± SD (days)	6.6 ± 3.9	10.1 ± 6.4	<0.001

EUS-GBD, endoscopic ultrasound-guided gallbladder drainage; N-ETGBD, rescue-endoscopic ultrasound-guided gallbladder drainage; PTGBD, percutaneous transhepatic gallbladder drainage; SD, standard deviation; T-ETGBD, rescue-percutaneous transhepatic gallbladder drainage; WBC, white blood cell.

## Data Availability

The dataset used during the current study is available from the corresponding author upon reasonable request.
